# Conserved roles of glucose in suppressing reactive oxygen species-induced cell death and animal survival

**DOI:** 10.18632/aging.102155

**Published:** 2019-08-12

**Authors:** Congrong Wang, Yinan Zhang, Fengwen Li, Yuehua Wei

**Affiliations:** 1 Shanghai East Hospital, Tongji University School of Medicine, Department of Endocrinology and Metabolic Disease, Translational Medical Center for Stem Cell Therapy, Shanghai, China; 2 Shanghai Jiao Tong University Affiliated Sixth People's Hospital, The Metabolic Diseases Biobank, Center for Translational Medicine, Shanghai Key Laboratory of Diabetes, Shanghai, China; 3 Shanghai Ninth People’s Hospital, Shanghai Jiao Tong University, School of Medicine, Shanghai, China

**Keywords:** aging, hyperglycemia, glucose, reactive oxygen species (ROS), SKN-1/Nrf2

## Abstract

Carbohydrate overconsumption increases blood glucose levels, which contributes to the development of various diseases including obesity and diabetes. It is generally believed that high glucose metabolism increases cellular reactive oxygen species (ROS) levels, damages insulin-secreting cells and leads to age-associated diabetic phenotypes. Here we find that in contrast, high glucose suppresses ROS production induced by paraquat in both mammalian cells and the round worm *C. elegans*. The role of glucose in suppressing ROS is further supported by glucose’s ability to alleviate paraquat’s toxicity on *C. elegans* development*.* Consistently, we find that the ROS-regulated transcription factor SKN-1 is inactivated by glucose. As a result, the ROS/SKN-1-dependent lifespan extension observed in paraquat-treated animals, mitochondrial respiration mutant *isp-1* and germline-less mutant *glp-1* are all suppressed by glucose. Our study reveals an unprecedented interaction of glucose with ROS, which could have significant impact on our current understanding of glucose- and ROS-related diseases.

## INTRODUCTION

High glycemic index (GI) diets are enriched in processed carbohydrates or sugars, which can be easily metabolized to glucose. High GI diets are known to cause hyperglycemia-associated diseases including obesity, type 2 diabetes and cardiovascular disease, even cancer [1–3]. Previous studies have convincingly shown that high blood glucose level is the major contributor to hyperglycemia-associated diseases [4, 5]. High blood glucose may directly affect metabolism, change body weight and alter hormone secretion, leading to a systemic deregulation of gene transcription [5, 6]. Despite rigorous research in the past few decades, our knowledge on how high glucose levels lead to these diseases remains limited.

To understand the basic mechanisms, researchers have examined the cellular and molecular changes after culturing cells in high glucose medium. It was found that high glucose treatment could lead to cellular dysfunction that might become irreversible over time, a process termed glucose toxicity [7, 8]. In addition, researchers also take advantage of genetic-tractable organisms such as *C. elegans* and fruit flies to investigate glucose toxicity [9, 10]. Similarly, high glucose can robustly increase the mortality rate and shorten lifespan [11–13]. High glucose uptake compromises the innate immune response to infection in *C. elegans* [14]. RNA sequencing in *C. elegans* demonstrates that in response to glucose, conserved genetic programs are induced to modulate several biological processes [15]. Together, research in *C. elegans* has begun to uncover new pathways underlying glucose toxicity in human.

Reactive oxygen species (ROS) play essential roles in modulating glucose metabolism and its downstream signaling [8, 16]. Accumulative data show that high glucose metabolisms can robustly increase cellular ROS levels, which are generated from altered mitochondrial functions [17, 18]. The ROS in turns causes damage to cellular macromolecules such as lipids, proteins and nucleic acids [16]. It is generally believed that the damage caused by high levels of ROS leads to dysregulation of cellular signaling and a variety of genetic pathways, which finally cause apoptosis and cell death [16, 19, 20].

Despite extensive research showing deleterious roles of ROS in glucose metabolisms and disease development, recently, a surprisingly effect of ROS in promoting health and delaying aging has also been reported [13, 21–24]. For example, *C. elegans* of mitochondrial respiration mutants such as *isp-1* and germline mutant *glp-1* have higher ROS levels compared with wild-type at young adult stage; such increase in ROS is necessary for their extended lifespan [22, 25, 26]. Interfering glucose metabolisms, which extends the lifespan of *C. elegans*, also increases endogenous ROS levels [13]. More directly, feeding *C. elegans* low levels of ROS-generating chemicals such as paraquat and juglone, robustly extends lifespan [25–27]. The beneficial ROS functions to induce protective transcriptional programs through stress response factors such as SKN-1, DAF-16, HIF-1 etc. [22, 25, 27]. Therefore, the role of ROS in aging and diseases is complex and remains controversial.

In this study, we use *Caenorhabditis elegans* as a model to dissect the early signal transduction of ROS in response to high glucose diet. We found that, glucose suppressed ROS production induced by paraquat. As a result, glucose alleviated the toxicity of paraquat on growth and development. Moreover, glucose suppressed the long lifespan of *C. elegans* caused by ROS. Molecularly, glucose inhibited the SKN-1-mediated detoxification pathway, which was necessary for ROS to extend life. We further showed that the suppression of ROS by glucose was conserved in mammalian cells. Our data provide several lines of evidence that glucose can suppress ROS generation to modulate health and lifespan.

## RESULTS

### Glucose mitigates the toxicity of paraquat on the development of *C. elegans*

**Figure 1:**
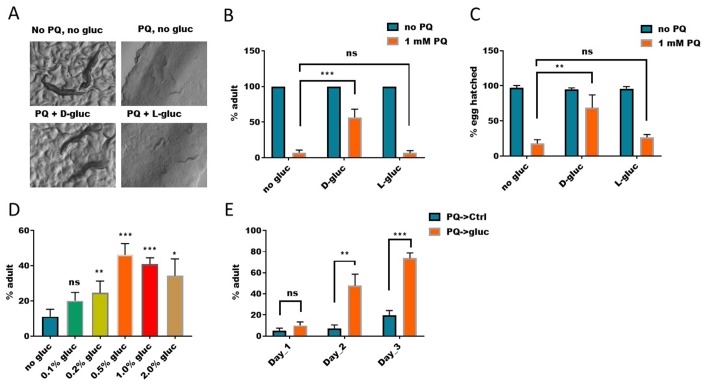
**Elevated glucose metabolism suppresses paraquat-induced development arrest.** (**A**) D-glucose but not L-glucose suppressed paraquat-induced development arrest. To synchronize eggs to the same development stage, eggs were collected from gravid worms within 2 hours. Synchronized eggs were then raised on nematode growth medium (NGM) agar plate with or without 1mM paraquat (PQ) and 1% glucose (D-gluc or L-gluc) for 4 days. Animals were imaged on plate with stereomicroscope. Representative images of 3 independent experiments were shown. (**B**) Quantification of animals developed to adulthood under paraquat and glucose treated conditions. *C. elegans* were treated as in (**A**) and percentage of animals reaching adulthood under indicated treatments were calculated and the mean values of 3 independent experiments (n>250) were plotted with error bars showing standard deviation (SD). P values were obtained by student’s t-test: ***, P<0.0005; ns, not significant. (**C**) D-glucose but not L-glucose suppressed paraquat-induced embryo lethality. Synchronized eggs from *C. elegans* were raised on NGM agar plate with or without paraquat (1mM) and 1% glucose (D-glucose or L-glucose). Hatching rate were calculated and the mean values of 3 independent experiments (n>300) were plotted with error bars showing the standard deviation. P values by student’s t-test: **, P<0.005; ns, not significant. (**D**) Dose-dependent effect of glucose on suppressing paraquat-induced developmental arrest. Synchronized eggs were raised on NGM agar plate with 1mM paraquat and indicated concentration of D-glucose for 4 days. The means of percentage of animals reaching adulthood from 3 independent experiments (n>300) were plotted, with error bars showing the standard deviation. P values by student’s t-test: *, P<0.05; **, P<0.005; *** P<0.0005; ns, not significant. (**E**) Glucose suppression of paraquat-induced toxicity occurred *in vivo*. Synchronized eggs from *C. elegans* were raised on NGM agar plate with 1mM paraquat without glucose for 2 days, then transferred to D-glucose plate without paraquat for indicated time points. The means of percentage of animals reaching adulthood from 3 experiments (n>250) were plotted, with error bars showing the standard deviation. P values by student’s t-test: **, P<0.005; *** P<0.0005; ns, not significant.

By serendipity, we found that glucose could partly rescue paraquat-induced toxicity in *C. elegans*. Paraquat is a widely used herbicide, which can generate high levels of cellular ROS through inhibiting Complex I of mitochondria. Paraquat has been known to implicate in many human diseases, including Alzheimer’s disease [28] and Parkinson disease [29]. We therefore wanted to further investigate the underlying signaling. Overproduction of cellular ROS has been thought to be the main reason accountable for paraquat’s cytotoxicity [30, 31]. Our experiment in *C. elegans* showed that, treatment of worms from hatching with 1mM of paraquat significantly inhibited growth and development, causing 95% of worms to be arrested at L2-L4 stages (Figure 1A and 1B). About 5% of worms reached adulthood but they produced only dead eggs (Figure 1C). Interestingly, however, if glucose was added, the percentage of the animals that reached adulthood increased to ~ 50% (Figure 1B) and the percentage of viable progenies also increased (Figure 1C). These rescuing effect of glucose on paraquat toxicity was not observed in non-metabolized L-glucose (Figure 1B and 1C), suggesting that an unknown glucose metabolite or metabolites is responsible for the suppression effect. The antagonizing effect of glucose on paraquat was dose dependent, as increasing glucose concentrations from 0.1% to 2% increased the number of worms reaching adulthood (Figure 1D). To rule out *in vitro* glucose-paraquat interaction, we first raised worms in medium containing 1mM paraquat from hatching to L2/L3 stage, then transferred to normal NG medium with and without glucose. We found that the suppressing effect of glucose on paraquat-induced developmental delay remained, albeit less robust (Figure 1E), suggesting that an in vivo product from glucose metabolism might affect paraquat to inhibit complex I of mitochondrial respiration chain.

### Glucose lowers ROS generation in *C. elegans*

**Figure 2:**
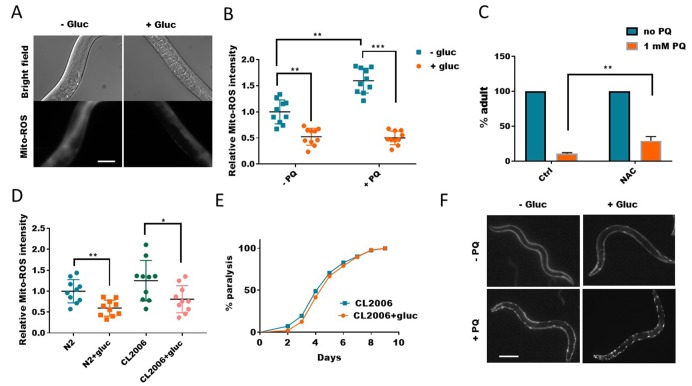
**Glucose suppresses mitochondrial reactive oxygen species (ROS) generation.** (**A**) Glucose suppressed paraquat-induced mitochondrial ROS. *C. elegans* were raised in the absence or presence of 0.5% glucose to L4/young adult stage then transferred to NGM agar plate containing 1mM paraquat for 24 hours. Animals were washed and incubated with Mitotracker-Red-Ros dye in M9 buffer for 2 hours. After extensive wash in M9 buffer, animals were raised on NGM agar plate for 4 hours then imaged by using fluorescence microscope. Experiments were repeated > 3 times. Representative images were shown. Scale bar: 60μm. (**B**) Glucose suppressed both WT and paraquat-induced mitochondrial ROS. Experiments were carried out as in (**A**). 10 images from multiple experiments were randomly selected and the fluorescence intensity (signal/area) was quantified by using ImageJ software. The average intensity of controls (no glucose and no paraquat) was defined as 1, to which all values were normalized. Error bars stand for standard deviation. P values by student’s t-test: **, P<0.005; *** P<0.0005. (**C**) Decreasing ROS alleviated paraquat from inhibiting development in *C. elegans*. Worms were raised on NGM agar plate containing 10mM antioxidant NAC (n-acetylcysteine) and 1mM paraquat (PQ) from hatching for 4 days. The mean percentage of animals that reached adulthood from 3 independent experiments (n>250) was plotted, with error bars showing standard deviation. P values by student’s t-test: **, P<0.005. (**D**) The elevated ROS levels in Aβ-expressing worms were decreased by glucose treatment. Wild-type (N2) and human Aβ-expressing worms (CL2006) were raised in the absence or presence of 0.5% glucose until day-1 adulthood. Worms were washed and stained with Mitotracker-Red-Ros dye as in (**A**). 10 images from 2 experiments were randomly selected and fluorescence intensity was quantified by using ImageJ software. Data were normalized to the mean intensity of N2 wild-type. Error bars stand for standard deviation. P values by student’s t-test: *, P<0.05; ** P<0.005. (**E**) Glucose suppression of ROS did not improve the paralysis of Aβ-expressing worms (CL2006). Animals were raised on NGM agar plate with and without 0.5% glucose from hatching at 25°C. Animals that no longer moved forward after gentle touch for 3 times were defined as paralyzed animals. Data were pooled from 3 independent experiments and plotted and statistically analyzed by using Prism software. P values by Log-rank test: not significant. (**F**) Glucose suppression of ROS exacerbated poly glutamine (polyQ35) aggregation in body wall muscle of *C. elegans*. Worms expressing polyQ35::YFP were raised in the absence or presence of 0.5% D-glucose to L4 stage, then transferred to NGM agar plate containing 1mM paraquat (PQ) for 2 days. Worms were picked and imaged with fluorescence microscope. Representative images of at least 3 independent experiments were shown. Scale bar: 200μm.

Since paraquat is known to generate ROS, which is widely believed to be the major contributing factor to paraquat’s cytotoxicity. One intriguing possibility is that glucose might prevent ROS generation induced by paraquat. To test this, we raised the worms with and without glucose to young adult, and treated worms with 1mM paraquat for 2 days. By using a dye that specifically stains mitochondrial ROS (MitoTracker-Red-ROS), we found that, paraquat-induced ROS was robustly reduced by glucose (Figure 2A and 2B). Glucose also slightly suppressed ROS generation in WT worms, suggesting a general role of glucose in reducing ROS *in vivo* (Figure 2B). Our experiment suggested that the rescuing effect in Figure 1 was probably a result of ROS suppression by glucose. To confirm this idea, we added ROS quencher NAC (N-acetyl-L-cysteine) to paraquat-treated worms. Similar to glucose, NAC also suppressed the delay in development caused by paraquat (Figure 2C), supporting the idea that glucose suppresses ROS generation to alleviate the toxicity of paraquat. It has been reported that glucose could suppress Aβ-induced paralysis in worms. However, there are also studies showing opposite results [32]. We tested if this was attributed to the suppression of ROS by glucose. We found that, consistently, glucose suppressed age-dependent ROS in both WT and Aβ-expressing worms CL2006 (Figure 2D). However, in several repeats, we did not observe any suppression of Aβ-induced paralysis (Figure 2E). We also tested if glucose could suppress aggregation of human huntingtin polyglutamine tract (polyQ35) in the body wall muscles. Strikingly, glucose only worsened the polyQ::YFP aggregation instead of alleviating it (Figure 2F). Our results suggest that glucose suppression of ROS is not involved in Aβ-induced paralysis and polyQ aggregation.

### Glucose inhibits SKN-1-mediated oxidative stress response.

**Figure 3:**
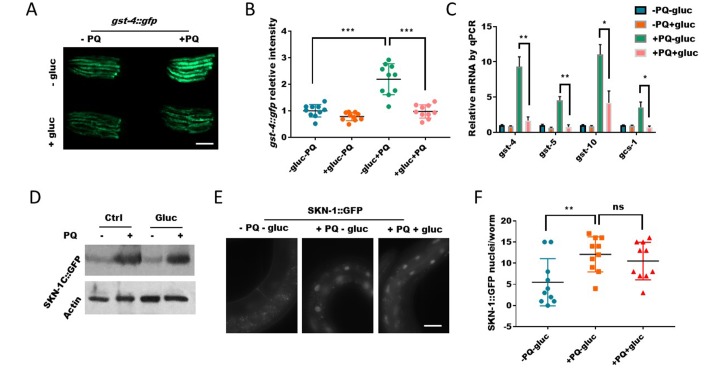
**Glucose signal inhibits SKN-1 activity in the nucleus.** (**A**) Glucose decreased the expression of SKN-1 target gene *gst-4* as revealed by a promoter GFP fusion (*gst-4::gfp*). Worms expressing *gst-4::gfp* were raised in the absence or presence of 0.5% D-glucose to L4 stage, then transferred to NGM agar plate with or without 1mM paraquat (PQ) for 2 days. Animals were picked and imaged with fluorescent microscope. Representative images of 3 independent experiments were shown. Scale bar: 400μm. (**B**) Quantification of GFP signal in individual worms in experiment described in (**A**). Worms (n=10) were randomly selected from 3 independent experiment and GFP intensity were quantified with ImageJ software. Data were normalized to the average value of control group (-gluc-PQ). Error bars stand for standard deviation (SD). P values by student’s t-test: *** P<0.0005. (**C**) Glucose decreased the expression of SKN-1 target genes revealed by real-time quantitative PCR (RT-qPCR). Glucose and paraquat treatments were the same as in (**A**). mRNA was extracted from animals with indicated treatments then reverse-transcribed to cDNA. The abundance of cDNA of indicated gene were quantified through RT-qPCR. The mean values of 2 independent experiments were plotted and analyzed by using Prism Software, with error bars showing standard deviation (SD). P values by student’s t-test: *, P<0.05; ** P<0.005. (**D**) The paraquat-induced SKN-1::GFP expression were not affected by glucose. Transgenic *C. elegans* expressing SKN-1::GFP were treated with glucose and paraquat as in (A). Worms (n>300) were homogenized and the whole lysates were used for western blot with specific antibodies against GFP or actin. (**E**) The nuclear localization of SKN-1::GFP upon paraquat treatment were not affected by glucose. Transgenic *C. elegans* expressing SKN-1::GFP were treated with glucose and paraquat as in (**A**), then imaged with fluorescence microscope. Representative image of multiple experiments (n>3) were shown. Scale bar: 60μm. (**F**) Quantification of experimental data from E. Animals were randomly selected from multiple experiments and the fluorescence intensities were quantified by ImageJ and plotted with Prism software, with error bars showing standard deviation (SD). P values by student’s t-test: ns, not significant; ** P<0.005.

Paraquat-induced ROS is known to activate, directly or indirectly, several transcription factors for oxidative stress response. Among these transcription factors, SKN-1 has a well-established function to mobilize detoxification program through gene transcription in order to restore the redox homeostasis under stress conditions [33]. Since glucose prevented paraquat from generate ROS (Figure 2), we would expect that glucose would also prevent paraquat from activating SKN-1. Consistent with this idea, we found that although the expression of SKN-1 target gene *gst-4::gfp* were increased by paraquat, such increase was suppressed in the presence of glucose (Figure 3A and 3B). By using quantitative PCR, we further demonstrated that several other SKN-1 target genes *gcs-1*, *gst-5*, *gst-10* were also suppressed by glucose (Figure 3C). SKN-1 is known to be activated through escaping the posttranslational degradation therefore accumulating in the nuclei [22, 33]. We tested if SKN-1 protein levels were affected by glucose. Contradictory to this established mode of regulation, despite the robust reduction in target gene expression, SKN-1 protein levels remained increased by paraquat regardless of glucose addition (Figure 3D), suggesting that glucose suppresses SKN-1 activity through novel mechanisms. We reasoned that glucose might decrease SKN-1 accumulation in the nucleus to activate gene transcription even though the overall protein levels remained unaffected. To this end, we examined the SKN-1::GFP translational reporter in an overexpression strain. Interestingly, we found that contradictory to this idea, paraquat-induced SKN-1 nuclear accumulation in the intestinal cells was not reduced (Figure 3E and 3F). Together, these results reveal the opposing effect of glucose and paraquat in modulating oxidative stress response and suggest a novel mechanism yet to be identified for glucose regulation of SKN-1.

### Glucose suppresses the beneficial effect of ROS on stress resistance, immunity and lifespan extension

**Figure 4:**
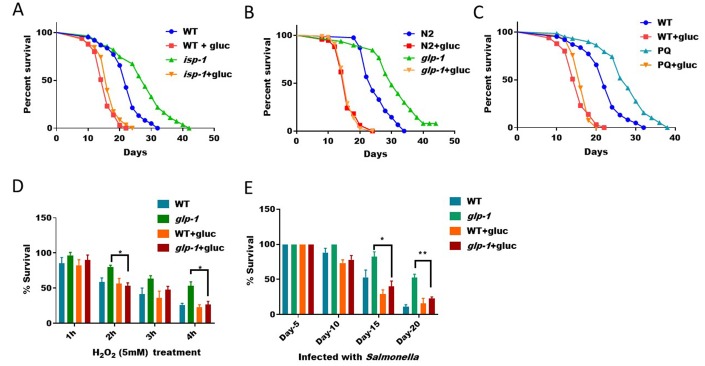
**ROS/SKN-1-dependent lifespan extension and stress resistance are suppressed by glucose supplementation.** (**A**) Glucose suppressed the extended lifespan of mitochondrial respiration mutant *isp-1*. N2 WT and *isp-1* mutant animals were raised at 20°C on NGM agar plate supplemented with or without 0.5% glucose from hatching. 50μM 5-Fluoro-2′-deoxyuridine (FUDR) was added at L4 stage to prevent progenies from growing. Survival was examined every 2 days until all animals died. Data were collected from 2 independent experiments (n> 140) and plotted and statistically analyzed with Prism software. P values by Log-rank test: *isp-1* vs. WT, P<0.0001; *isp-1*+glucose vs. WT+glucose: not significant. (**B**) Glucose suppressed the extended lifespan of germline-less mutant *glp-1*. Lifespan assay of N2 WT and *glp-1* mutant animals were conducted as in (**A**) except that worms where raised at 25°C from L1 for 24 hours. Data from 2 independent trials (n>120) were pooled and plotted by using Prism software. P values by Log-rank test: *glp-1* vs. WT, P<0.0001; *glp-1*+glucose vs. WT+glucose: not significant. (**C**) Glucose suppressed the extended lifespan of paraquat-treated *C. elegans*. Synchronized eggs were raised on control NGM plate, plates with either 1mM paraquat or 0.5% glucose, or both. Young adult worms were transferred to new plates for lifespan assay similar to (**A**). Data from 2 independent trials (n>120) were pooled and plotted by using Prism software. P values by Log-rank test: PQ vs. WT, P<0.0001; PQ+glucose vs. WT+glucose: not significant. (**D**) The hydrogen peroxide resistance of germline-less mutant (*glp-1*) was abrogated by glucose. N2 WT and the temperature sensitive *glp-1* mutant animals growing with or without 0.5% glucose were raised under non-permissive temperature (25°C) from L1 stage for 24 hours, then shifted back to 20°C until day-1 adulthood. Animals were then incubated with 5mM of H_2_O_2_ in water for indicated time points. Survival rates of the means of 3 independent experiments were plotted for each time point, with error bars showing standard deviation (SD). P value by student’s t-test: *, P<0.05. (**E**) Resistance to infection of germline-less mutant (*glp-1*) were abrogated by glucose. N2 WT and the temperature sensitive *glp-1* mutant animals growing with and without 0.5% glucose were raised at non-permissive temperature (25°C) from L1 stage for 24 hours, then shifted back to 20°C. Animals were then fed *Salmonella typhimurium* on agar plate for indicated time points and survival rate of the mean values of 3 independent experiments were plotted, with error bars indicating standard deviation (SD). P value by student’s t-test: *, P<0.05; **, P<0.005.

Activation of SKN-1-mediated antioxidant program is known to restore intracellular redox homeostasis. In *C. elegans*, several long-lived mutants, such as mitochondrial respiration mutant (*isp-1*) and germline mutant (*glp-1*), depend on ROS to activate SKN-1-mediated antioxidant program. The activation of SKN-1 by ROS improves resistance to oxidative stress, innate immunity and other detoxification process, which together contribute to the lifespan extension in these animals [33]. Since our previous results show that high glucose diet can suppress ROS generation (Figure 1), we predicted that glucose would also prevent the longevity of these mutant animals. To test this prediction, we fed long-lived *isp-1* and *glp-1* mutants and control animals with and without glucose. We found that, in the presence of glucose, the extended lifespan of these animals was robustly suppressed (Figure 4A and 4B). In addition, treating worms with low levels of ROS generator paraquat is known to extend lifespan [12, 26, 34]. However, such lifespan increase was cancelled in glucose diet (Figure 4C). Therefore, glucose antagonizes the beneficial effect of ROS on extending *C. elegans’* lifespan. Since oxidative stress resistance and improved immunity are features of these long-lived animals, we further tested if such features were suppressed by glucose. Consistent with earlier results [22], *glp-1* mutant was resistant to hydrogen peroxide, however, in the presence of glucose, such resistance was blocked (Figure 4D). Similarly, *glp-1* mutant was resistant to killing by *Salmonella*, however, such resistance was also blocked by glucose addition (Figure 4E).

### The suppression of ROS by glucose is conserved in mammalian cells

**Figure 5:**
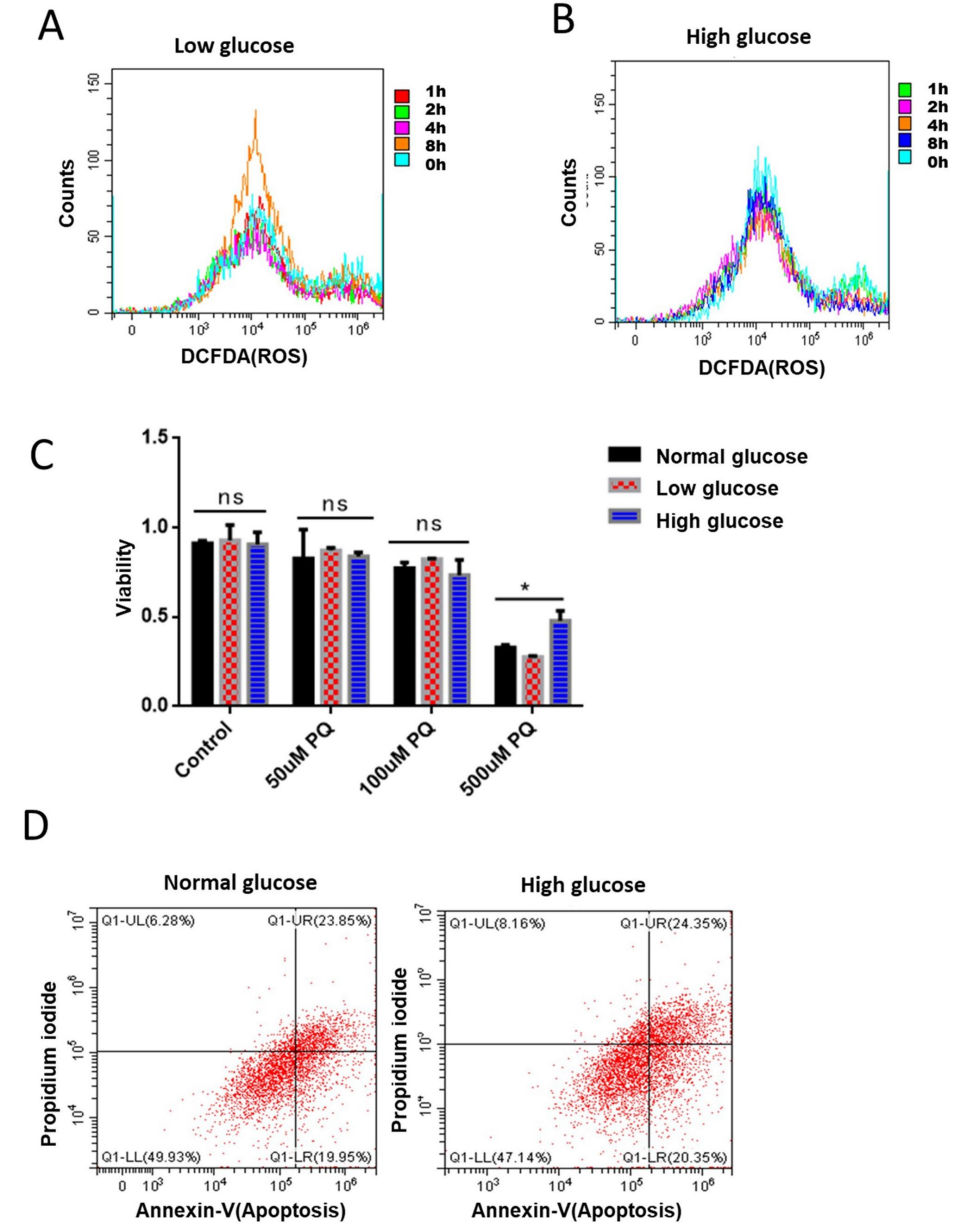
**Glucose suppression of ROS is conserved in mammalian cells.** (**A**) Short term rapamycin treatment increased intracellular ROS levels. Mouse embryonic fibroblasts (NIH3T3) cultured in medium supplemented with 1g/L glucose were treated with 100nM rapamycin for 1, 2, 4, and 8 hours. ROS levels were measured by staining cells with 2,7′–dichlorofluorescin diacetate (DCFDA), followed by flow cytometry analysis. (**B**) High glucose suppressed rapamycin-induced ROS in mouse embryonic fibroblasts. Mouse embryonic fibroblasts (NIH3T3) cultured in medium supplemented with 8g/L glucose were treated with 100nM rapamycin for 1, 2, 4, and 8 hours. ROS levels were measured by staining cells with 2,7′–dichlorofluorescin diacetate (DCFDA), followed by flow cytometry analysis. (**C**) High glucose suppressed ROS-induced cell death in mouse embryonic fibroblasts. NIH3T3 cells maintained in low (1g/L), normal (4g/L) and high glucose (8g/L) were treated with indicated concentrations of ROS-generator paraquat for 24 hours. Cell viability was detected by Cell Counting Kit-8 (CCK-8) assay. Means of 3 independent experiments were plotted with error bars showing the standard deviation. P value by student’s t-test: *, P<0.05. (**D**) Apoptosis was not significantly repressed by glucose. NIH3T3 cells maintained in normal (4g/L) and high glucose (8g/L) were treated 500μM ROS generator paraquat for 24 hours to induce cell apoptosis. Cell viability was measured by Propidium iodide (PI) staining and apoptosis were detected by Annexin V-FITC followed by flow cytometry analysis.

The opposing effect of glucose and ROS in *C. elegans*’ development and lifespan may suggest significant mechanisms in modulating ROS- and glucose-related disease in human. We therefore were interested to know whether glucose-ROS interaction was conserved in higher organisms, by testing if glucose would antagonize ROS generation in mammalian cells. Long term rapamycin treatment has been reported to suppress ROS production [35], which involves activation of Nrf2 anti-oxidation program [36]. We reasoned that rapamycin would increase ROS first to activate Nrf2, therefore induce transcriptional programs to reduce overall ROS. To test this, we treated NIH3T3 cells with 100nM rapamycin for 1, 2, 4 and 8 hours, then examined the changes in ROS production. Indeed, rapamycin treatment for 8 hours increased significantly the production of ROS (Figure 5A). We next asked if glucose would suppress the ROS induction in rapamycin-treated mammalian cells. By repeating the same experiments in the presence of glucose, we found that glucose indeed prevented rapamycin from inducing ROS (Figure 5B).

Elevated ROS levels could damage macromolecules such as DNA, RNA, proteins and lipids, finally leading to cell death. Since our data has established the role of glucose in suppressing ROS and ROS-related physiology in *C. elegans*, we wondered if glucose would suppress ROS-induced cell death in mammalian cells. To this end, we treated NIH3T3 cells that had been maintained in low (1g/L), normal (4g/L) and high glucose (8g/L) with various concentrations of the ROS-generator paraquat for 24 hours. Our results showed that 500nM of paraquat robustly decreased the survival of NIH3T3 cells. Interestingly, the killing effect of paraquat can be significantly mitigated by culturing cells in high glucose medium (Figure 5C). Paraquat’s killing effect has been attributed to its effect on inducing robust apoptosis [37], we therefore examined if glucose’s role in suppressing paraquat toxicity was through apoptosis. By using FTIC-conjugated Annexin V as an indicator of apoptosis, we found no suppression of apoptosis by glucose, suggesting that glucose effect on protecting survival under high ROS condition involves new mechanisms (Figure 5D).

## DISCUSSION

Dysregulation of glucose metabolism is known to cause age-associated physiological decline and diseases in human [1–3]. Overconsumption of glucose is linked to type 2 diabetes but dissecting the underlying mechanisms remains challenging. By examining glucose effect on *C. elegans* development and aging, we report here that glucose suppresses ROS production, reduces paraquat-induced toxicity and benefits health. Consistently, glucose also suppresses ROS-dependent lifespan extension. Since glucose has been widely known to increase ROS in previous study [11, 18, 38, 39], our finding is unexpected. In addition, we show that this unexpected regulation is conserved in mammalian cell lines. Our results are supported by a recent metabolomic profiling in hepatocellular carcinoma showing that increased glucose metabolism is associated with reduced anti-oxidative metabolites and poor survival rate [40]. Therefore, further investigation into the novel and conserved mechanisms underlying glucose suppression of ROS might have significant relevance to human disease including cancer and hyperglycemia.

**Figure 6:**
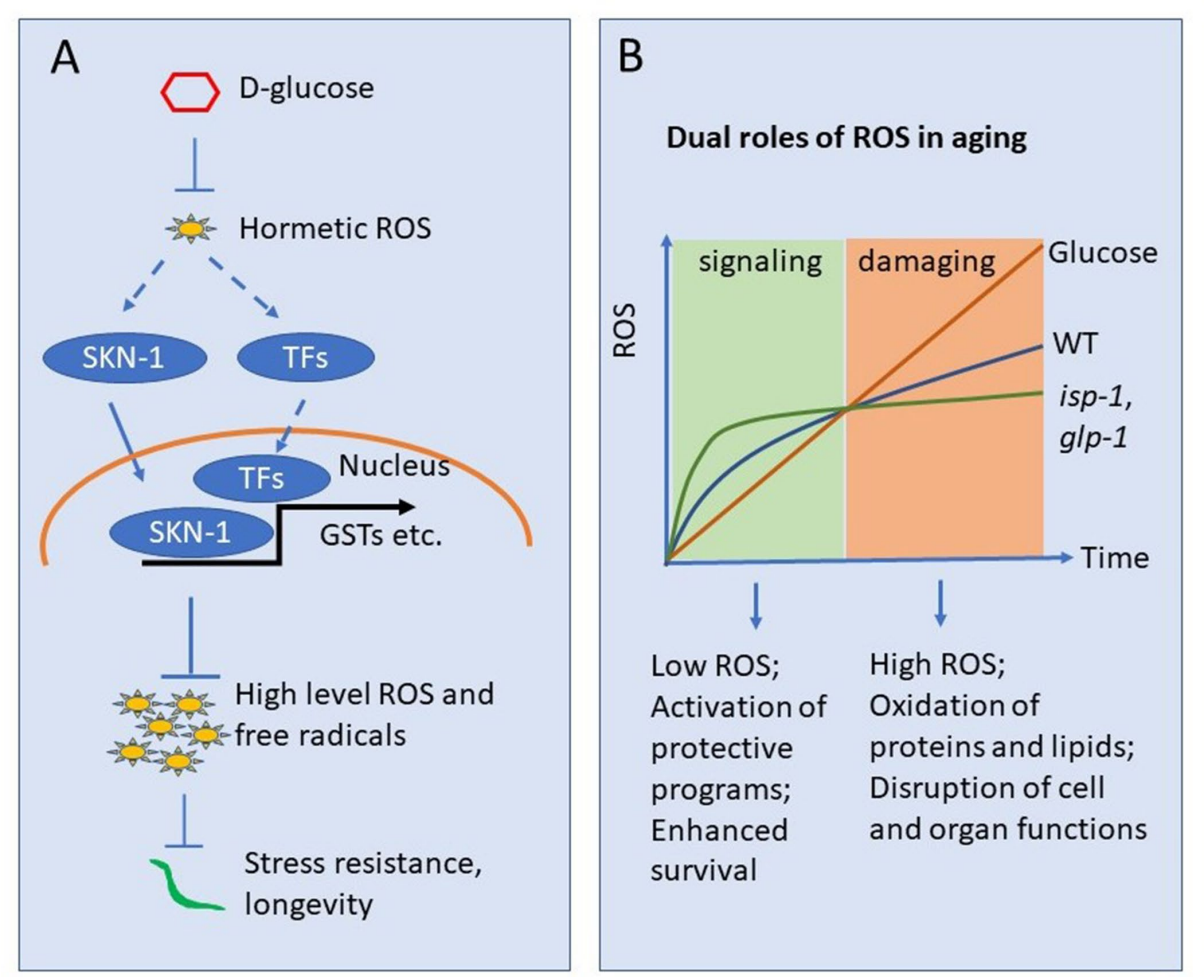
**Working model showing glucose suppression of ROS hormesis to accelerate aging.** (**A**) Oxidative stress at early stage may induce hormetic ROS, which in turn wages oxidative stress response by activating transcription factors (TFs) such as SKN-1. Glucose can suppress such hormetic ROS, therefore inhibiting TFs such as SKN-1. The suppression of hormetic ROS by glucose leads to uncontrolled elevation of ROS later in life and organismal death. (**B**) ROS accumulates as organisms age. Early in life, low levels of ROS serve as signaling to induce oxidative stress response, which in turns slower oxidative damage later in life. The ROS in early life serves a hormetic signal. *glp-1* and *isp-1* induce hormetic ROS and live longer than WT animals. However, glucose suppression of such hormetic ROS accelerates ROS accumulation and blocks lifespan extension in these long-lived mutants.

Previous results have shown abundantly that elevated glucose metabolism increases ROS through mitochondria [41]. However, these results are mostly based on *in vitro* studies on cell lines or long-term glucose treatment. In contrast, our short-term *in vivo* studies in *C. elegans* and mammalian cell lines demonstrate that glucose suppresses ROS (Figures 2A, 5A and 5B), which is further confirmed by the antagonizing effect of glucose and ROS on development and survival (Figure 1). The glucose suppression of ROS inhibits the ROS-induced oxidative stress response (Figure 3), likely through SKN-1, a Nrf2 homolog in *C. elegans*. As Nrf2/SKN-1 activation is known to activate transcription programs to defense against high levels of free radicals [42, 43], glucose suppression of SKN-1 could finally increase free radical levels. Indeed, we confirm that longer treatment of glucose increases Dihydroethidium (DHE)-stained ROS (Supplementary Figure 1). Therefore, our result is consistent with previous studies showing that long term glucose treatment increases ROS [11, 18, 38, 41]. Note that glucose may not regulate SKN-1, as we did not observe any change in SKN-1 protein levels. However, SKN-1 posttranslational modification could be modified and regulated by glucose. Alternatively, glucose could engage other factors as co-regulator for SKN-1 to repress target genes transcription (Figure 6A).

Recently, the idea of ROS hormesis has been added to mitochondrial aging theory to explain several surprising observations: various ROS generators, including paraquat and juglone, invariably extend lifespan [21, 24, 44, 45]. Consistently, mutations that extend lifespan, including *isp-1* and *glp-1*, show unexpectedly increased levels of ROS early in adulthood [12, 22, 23, 26, 46]. This is obviously contrasting to the mitochondrial theory of aging, which predicts that ROS generating events, by chemicals or genetic modulations, should reduce rather than increase lifespan. A reasonable explanation for the discrepancy is ROS hormesis: early induction of ROS at moderate levels can induce protecting programs to defense against high levels of ROS, which ultimately leads to extended lifespan (Figure 6A). Indeed, our data is consistent with the hormesis theory in that the glucose initially suppresses ROS, preventing SKN-1-dependent antioxidant programs, then increases ROS and shortens lifespan.

Another key point of the hormesis theory of aging is the dual role of ROS [47]. Although largely neglected in the past, the long-held belief that ROS acts passively to promote aging and related diseases has been recently challenged, as cumulating experimental evidence shows that ROS could also serve positively to improve health and extend lifespan [44, 48]. In our case, glucose suppression of ROS could disrupt the early spike of ROS in Figure 6B (which is beneficial because it can induce hormesis), therefore initiate aging signals that can propagate to later life so as to accelerate the aging process and disease development. Since ROS is involved in a variety of signaling pathways implicated in human diseases [49, 50], our studies could encourage further investigations that may lead to clinical interventions and cures.

Glucose not only prevents the ROS-dependent lifespan extension, but also robustly reduces the lifespan of wild-type animals [12, 13]. It is possible that ROS hormesis also occurs in wild-type, repression of which can lead to lifespan shortening. Indeed, we found that glucose also reduced ROS in wild-type animals as well (Figure 2B) and suppressed SKN-1 target gene transcription (Figure 3A and 3B). Therefore, at least part of the lifespan reduction in wild-type animals could be attributed to glucose suppression of ROS and SKN-1. However, whether ROS hormesis occurs in wild-type animals and how much it can explain glucose’s effect on lifespan reduction remains unknown. Future studies focusing on longitudinal analysis will give better answers to these questions.

How does glucose suppress ROS? We have proven that the glucose does not simply prevent the paraquat effect in the medium (Figure 1E). Glucose, through cytosolic glycolysis and mitochondrial TCA cycle, generates energy in the form of ATP for cellular activities. ROS is produced as a byproduct of ATP generation through TCA cycle in the mitochondria, but not glycolysis in the cytosol. It is known that glycolysis and TCA could have reciprocal repression/interaction [51–53], likely as a mechanism to avoid overproduction of deleterious ROS. In our study, high glucose metabolism in the short term could favor ATP generation through glycolysis, therefore feeding back to slow down mitochondrial TCA cycle hence ROS generation. Alternatively, the elevated glycolysis could also lead to glucose metabolism through pentose phosphate pathway (PPP), which would generate NADPH to scavenge ROS surplus [54, 55]. It is likely that one or a few of the glucose metabolites serve as signaling molecules to balance glucose metabolism and ROS production. Better understanding of the detailed regulatory mechanisms requires future research through global profiling of metabolites in appropriate model systems.

## MATERIALS AND METHODS

Detailed experimental procedures can be found in Supplementary Information.

### Strains, cell line and culture medium

*C. elegans* strains were maintained on nematode grow medium (NGM) agar plate seeded with OP-50 bacteria. Glucose was added before pouring agar plate. The genotypes of *C. elegans* used in this study are shown in Supplementary Information, Supplementary Table 1. NIH3T3 cells were maintained in DMEM (Sigma) supplemented with 10% FBS (Gbico) and 100μg/mL Penicillin-Streptomycin Solution (Gbico) at 37 °C in a humidified atmosphere containing 5% CO2.

### Drug treatment

NAC (n-acetylcysteine, Sigma) and paraquat (methyl viologen dichloride, Sigma) were added to agar plates at the indicated concentrations 1 day before use. Except for development assay, where paraquat was initiated from synchronized egg, animals were treated with paraquat from L4/young adult stage. NAC treatment was initiated from synchronized eggs for all experiments. Hydrogen peroxide (H_2_O_2_) treatment was conducted in liquid by incubating L4/young adult worms with 5mM H_2_O_2_ for 1–4 hours. For lifespan assay, 50uM FUDR (5-Fluoro-2′-deoxyuridine) was added to agar plate at the late L4 stage (of control as well as experimental animals) to inhibit reproduction.

### ROS detection

Mitochondrial ROS was detected using the mitochondrial-localized ROS sensor Mitotracker (MitoTracker® Red CMXRos). Animals with different treatments were washed with M9 buffer extensively to remove bacteria food. Animals were then incubated with 5μM Mitoracker-Red-Ros in M9 buffer for 2 hours with gentle shaking to allow uptake of Mito-Tracker dye. Animals were then washed extensively to remove excessive MitoTracker dye and raised again on NGM agar plate for at least 4 hours before imaging with fluorescent microscope. For ROS detection in mammalian cells, NIH3T3 cells were stained with 10 μM of Dichloro-dihydro-fluorescein diacetate (DCFH-DA) in serum-free RPMI-1640 medium at 37 °C for 30min, followed by flow cytometry detection.

### Western blot

Worm were washed from plates with ice-cold M9 buffer extensively to remove bacteria, then sonicated in lysis buffer (50 mM HEPES, pH 7.4, 1 mM EGTA, 1 mM MgCl2, 100 mM KCl, 10% glycerol, 0.5% NP-40, 2mM PMSF, Roche protease Complete inhibitor cocktail and phosSTOP tablet). Whole lysate was subjected to SDS-PAGE and transferred to PVDF membrane. Membranes were blocked in 5% non-fat milk and probed with anti-GFP (Abcam, ab32146) and anti-actin antibodies (Abcam, ab14128) at 1000X dilution, followed by anti-rabbit and anti-mouse HRP-conjugated secondary antibodies at 10,000X dilution, then detected by enhanced chemiluminescence (ECL). Detailed procedures have also been described before [56, 57].

### Real-time quantitative PCR

Worms were washed with ice-cold M9 buffer from plates and total mRNA were extracted by Trizol method. mRNA was reverse-transcribed using QIAGEN One-Step RT-PCR Kit to obtain cDNA. Quantitative PCR was performed using SYBR Green 2X Mater Mix (Applied Biosystems). Gene expression levels were normalized to actin (*ACT1*) and expressed as fold changes to that of the wild-type. Primers have been published before [14], which are also listed in Supplementary Information, Supplementary Table 2.

### *C. elegans* paralysis assay

Gravid hermaphrodites expressing human Aβ(1-42) in body-wall muscles were allowed to lay eggs to new plates for 2 hour to collect synchronized progenies. Gravid worms were removed and plates were incubated at 25°C. Worms were scored for paralysis at the indicated time points starting from day-1 of adulthood. Worms that failed to move when touched with a platinum wire were scored as “paralyzed”.

### Pathogen infection and survival measurement

*Salmonella typhimurium* infection has been described before [58]. Log phase *Salmonella culture* was spun down and plated on NGM agar plates, to which L4/young adult worms were added. After infection for 24 hours, worms were transferred back to NGM agar plates seeded with non-pathogenic OP-50. Infected animals were examined for death every other day and data were plotted and statistically analyzed by using Graphpad Prism software.

### Lifespan assays

Gravid worms were transferred to assay plates to collect synchronized eggs. 50uM FUDR (5-Fluoro-2′-deoxyuridine) was added to agar plate at the late L4 stage to inhibit reproduction. The number of live and dead worms was recorded every other day from day 8. Death was defined by lack of any visible movement for 5 seconds after touching the tail. Worms that ruptured or crawled off the plate were censored. To inactivate *glp-1*, wild-type and mutant worms at L1 stage were treated with non-permissive temperature (25°C) for 24 hours then shifted to 20 °C for the rest of the experiment. Data and statistics are also shown in Supplementary Information, Supplementary Tables 3–5.

## Supplementary Material

Supplementary Information

Supplementary Figure 1

Supplementary Tables

## References

[1] Aston LM. Glycaemic index and metabolic disease risk. Proc Nutr Soc. 2006; 65:125–34. 10.1079/PNS2005485 16441952

[2] Venn BJ, Green TJ. Glycemic index and glycemic load: measurement issues and their effect on diet-disease relationships. Eur J Clin Nutr. 2007 (Suppl 1); 61:S122–31. 10.1038/sj.ejcn.1602942 17992183

[3] Duan W, Shen X, Lei J, Xu Q, Yu Y, Li R, Wu E, Ma Q. Hyperglycemia, a neglected factor during cancer progression. Biomed Res Int. 2014; 2014:461917. 10.1155/2014/461917 24864247 PMC4016871

[4] Kharroubi AT, Darwish HM. Diabetes mellitus: the epidemic of the century. World J Diabetes. 2015; 6:850–67. 10.4239/wjd.v6.i6.850 26131326 PMC4478580

[5] De Rosa S, Arcidiacono B, Chiefari E, Brunetti A, Indolfi C, Foti DP. Type 2 Diabetes Mellitus and Cardiovascular Disease: Genetic and Epigenetic Links. Front Endocrinol (Lausanne). 2018; 9:2. 10.3389/fendo.2018.00002 29387042 PMC5776102

[6] Alcántar-Fernández J, Navarro RE, Salazar-Martínez AM, Pérez-Andrade ME, Miranda-Ríos J. Caenorhabditis elegans respond to high-glucose diets through a network of stress-responsive transcription factors. PLoS One. 2018; 13:e0199888. 10.1371/journal.pone.0199888 29990370 PMC6039004

[7] Robertson RP, Harmon J, Tran PO, Tanaka Y, Takahashi H. Glucose toxicity in beta-cells: type 2 diabetes, good radicals gone bad, and the glutathione connection. Diabetes. 2003; 52:581–87. 10.2337/diabetes.52.3.581 12606496

[8] Kawahito S, Kitahata H, Oshita S. Problems associated with glucose toxicity: role of hyperglycemia-induced oxidative stress. World J Gastroenterol. 2009; 15:4137–42. 10.3748/wjg.15.4137 19725147 PMC2738809

[9] Lee D, Jeong DE, Son HG, Yamaoka Y, Kim H, Seo K, Khan AA, Roh TY, Moon DW, Lee Y, Lee SJ. SREBP and MDT-15 protect C. elegans from glucose-induced accelerated aging by preventing accumulation of saturated fat. Genes Dev. 2015; 29:2490–503. 10.1101/gad.266304.115 26637528 PMC4691952

[10] Galenza A, Hutchinson J, Campbell SD, Hazes B, Foley E. Glucose modulates Drosophila longevity and immunity independent of the microbiota. Biol Open. 2016; 5:165–73. 10.1242/bio.015016 26794610 PMC4823985

[11] Schlotterer A, Kukudov G, Bozorgmehr F, Hutter H, Du X, Oikonomou D, Ibrahim Y, Pfisterer F, Rabbani N, Thornalley P, Sayed A, Fleming T, Humpert P, et al. C. elegans as model for the study of high glucose-mediated life span reduction. Diabetes. 2009; 58:2450–56. 10.2337/db09-0567 19675139 PMC2768179

[12] Lee SJ, Murphy CT, Kenyon C. Glucose shortens the life span of C. elegans by downregulating DAF-16/FOXO activity and aquaporin gene expression. Cell Metab. 2009; 10:379–91. 10.1016/j.cmet.2009.10.003 19883616 PMC2887095

[13] Schulz TJ, Zarse K, Voigt A, Urban N, Birringer M, Ristow M. Glucose restriction extends Caenorhabditis elegans life span by inducing mitochondrial respiration and increasing oxidative stress. Cell Metab. 2007; 6:280–93. 10.1016/j.cmet.2007.08.011 17908557

[14] Li L, Chen Y, Chenzhao C, Fu S, Xu Q, Zhao J. Glucose negatively affects Nrf2/SKN-1-mediated innate immunity in *C. elegans* Aging (Albany NY). 2018; 10:3089–103. 10.18632/aging.101610 30442878 PMC6286829

[15] Garcia AM, Ladage ML, Dumesnil DR, Zaman K, Shulaev V, Azad RK, Padilla PA. Glucose induces sensitivity to oxygen deprivation and modulates insulin/IGF-1 signaling and lipid biosynthesis in Caenorhabditis elegans. Genetics. 2015; 200:167–84. 10.1534/genetics.115.174631 25762526 PMC4423361

[16] Volpe CM, Villar-Delfino PH, Dos Anjos PM, Nogueira-Machado JA. Cellular death, reactive oxygen species (ROS) and diabetic complications. Cell Death Dis. 2018; 9:119. 10.1038/s41419-017-0135-z 29371661 PMC5833737

[17] Yu T, Robotham JL, Yoon Y. Increased production of reactive oxygen species in hyperglycemic conditions requires dynamic change of mitochondrial morphology. Proc Natl Acad Sci USA. 2006; 103:2653–58. 10.1073/pnas.0511154103 16477035 PMC1413838

[18] Yu T, Jhun BS, Yoon Y. High-glucose stimulation increases reactive oxygen species production through the calcium and mitogen-activated protein kinase-mediated activation of mitochondrial fission. Antioxid Redox Signal. 2011; 14:425–37. 10.1089/ars.2010.3284 20518702 PMC3025178

[19] Allen DA, Yaqoob MM, Harwood SM. Mechanisms of high glucose-induced apoptosis and its relationship to diabetic complications. J Nutr Biochem. 2005; 16:705–13. 10.1016/j.jnutbio.2005.06.007 16169208

[20] Cai L, Li W, Wang G, Guo L, Jiang Y, Kang YJ. Hyperglycemia-induced apoptosis in mouse myocardium: mitochondrial cytochrome C-mediated caspase-3 activation pathway. Diabetes. 2002; 51:1938–48. 10.2337/diabetes.51.6.1938 12031984

[21] Wei Y, Zhang YJ, Cai Y, Xu MH. The role of mitochondria in mTOR-regulated longevity. Biol Rev Camb Philos Soc. 2015; 90:167–81. 10.1111/brv.12103 24673778

[22] Wei Y, Kenyon C. Roles for ROS and hydrogen sulfide in the longevity response to germline loss in Caenorhabditis elegans. Proc Natl Acad Sci USA. 2016; 113:E2832–41. 10.1073/pnas.1524727113 27140632 PMC4878494

[23] Yee C, Yang W, Hekimi S. The intrinsic apoptosis pathway mediates the pro-longevity response to mitochondrial ROS in C. elegans. Cell. 2014; 157:897–909. 10.1016/j.cell.2014.02.055 24813612 PMC4454526

[24] Zhang XS, Wang T, Lin XW, Denlinger DL, Xu WH. Reactive oxygen species extend insect life span using components of the insulin-signaling pathway. Proc Natl Acad Sci USA. 2017; 114:E7832–40. 10.1073/pnas.1711042114 28847950 PMC5604040

[25] Lee SJ, Hwang AB, Kenyon C. Inhibition of respiration extends C. elegans life span via reactive oxygen species that increase HIF-1 activity. Curr Biol. 2010; 20:2131–36. 10.1016/j.cub.2010.10.057 21093262 PMC3058811

[26] Yang W, Hekimi S. A mitochondrial superoxide signal triggers increased longevity in Caenorhabditis elegans. PLoS Biol. 2010; 8:e1000556. 10.1371/journal.pbio.1000556 21151885 PMC2998438

[27] Heidler T, Hartwig K, Daniel H, Wenzel U. Caenorhabditis elegans lifespan extension caused by treatment with an orally active ROS-generator is dependent on DAF-16 and SIR-2.1. Biogerontology. 2010; 11:183–95. 10.1007/s10522-009-9239-x 19597959

[28] Manning-Bog AB, McCormack AL, Li J, Uversky VN, Fink AL, Di Monte DA. The herbicide paraquat causes up-regulation and aggregation of alpha-synuclein in mice: paraquat and alpha-synuclein. J Biol Chem. 2002; 277:1641–44. 10.1074/jbc.C100560200 11707429

[29] Berry C, La Vecchia C, Nicotera P. Paraquat and Parkinson’s disease. Cell Death Differ. 2010; 17:1115–25. 10.1038/cdd.2009.217 20094060

[30] Suntres ZE. Role of antioxidants in paraquat toxicity. Toxicology. 2002; 180:65–77. 10.1016/S0300-483X(02)00382-7 12324200

[31] Abdollahi M, Ranjbar A, Shadnia S, Nikfar S, Rezaie A. Pesticides and oxidative stress: a review. Med Sci Monit. 2004; 10:RA141–47. 15173684

[32] Yang Y, Wu Y, Zhang S, Song W. High glucose promotes Aβ production by inhibiting APP degradation. PLoS One. 2013; 8:e69824. 10.1371/journal.pone.0069824 23894546 PMC3720941

[33] Blackwell TK, Steinbaugh MJ, Hourihan JM, Ewald CY, Isik M. SKN-1/Nrf, stress responses, and aging in Caenorhabditis elegans. Free Radic Biol Med. 2015; 88:290–301. 10.1016/j.freeradbiomed.2015.06.008 26232625 PMC4809198

[34] Cypser JR, Johnson TE. Multiple stressors in Caenorhabditis elegans induce stress hormesis and extended longevity. J Gerontol A Biol Sci Med Sci. 2002; 57:B109–14. 10.1093/gerona/57.3.B109 11867647

[35] Martínez-Cisuelo V, Gómez J, García-Junceda I, Naudí A, Cabré R, Mota-Martorell N, López-Torres M, González-Sánchez M, Pamplona R, Barja G. Rapamycin reverses age-related increases in mitochondrial ROS production at complex I, oxidative stress, accumulation of mtDNA fragments inside nuclear DNA, and lipofuscin level, and increases autophagy, in the liver of middle-aged mice. Exp Gerontol. 2016; 83:130–38. 10.1016/j.exger.2016.08.002 27498120

[36] Lerner C, Bitto A, Pulliam D, Nacarelli T, Konigsberg M, Van Remmen H, Torres C, Sell C. Reduced mammalian target of rapamycin activity facilitates mitochondrial retrograde signaling and increases life span in normal human fibroblasts. Aging Cell. 2013; 12:966–77. 10.1111/acel.12122 23795962 PMC5559196

[37] Chen YW, Yang YT, Hung DZ, Su CC, Chen KL. Paraquat induces lung alveolar epithelial cell apoptosis via Nrf-2-regulated mitochondrial dysfunction and ER stress. Arch Toxicol. 2012; 86:1547–58. 10.1007/s00204-012-0873-8 22678742

[38] Abuarab N, Munsey TS, Jiang LH, Li J, Sivaprasadarao A. High glucose-induced ROS activates TRPM2 to trigger lysosomal membrane permeabilization and Zn^2+^-mediated mitochondrial fission. Sci Signal. 2017; 10:10. 10.1126/scisignal.aal4161 28765513

[39] Asmat U, Abad K, Ismail K. Diabetes mellitus and oxidative stress-A concise review. Saudi Pharm J. 2016; 24:547–53. 10.1016/j.jsps.2015.03.013 27752226 PMC5059829

[40] Cassim S, Raymond VA, Lacoste B, Lapierre P, Bilodeau M. Metabolite profiling identifies a signature of tumorigenicity in hepatocellular carcinoma. Oncotarget. 2018; 9:26868–83. 10.18632/oncotarget.25525 29928490 PMC6003570

[41] Bonnefont-Rousselot D. Glucose and reactive oxygen species. Curr Opin Clin Nutr Metab Care. 2002; 5:561–68. 10.1097/00075197-200209000-00016 12172481

[42] Kim J, Keum YS. NRF2, a Key Regulator of Antioxidants with Two Faces towards Cancer. Oxid Med Cell Longev. 2016; 2016:2746457. 10.1155/2016/2746457 27340506 PMC4909917

[43] Schmidlin CJ, Dodson MB, Madhavan L, Zhang DD. Redox regulation by NRF2 in aging and disease. Free Radic Biol Med. 2019; 134:702–07. 10.1016/j.freeradbiomed.2019.01.016 30654017 PMC6588470

[44] Ristow M, Schmeisser K. Mitohormesis: Promoting Health and Lifespan by Increased Levels of Reactive Oxygen Species (ROS). Dose Response. 2014; 12:288–341. 10.2203/dose-response.13-035.Ristow 24910588 PMC4036400

[45] Ludovico P, Burhans WC. Reactive oxygen species, ageing and the hormesis police. FEMS Yeast Res. 2014; 14:33–39. 10.1111/1567-1364.12070 23965186 PMC4332618

[46] Hwang AB, Ryu EA, Artan M, Chang HW, Kabir MH, Nam HJ, Lee D, Yang JS, Kim S, Mair WB, Lee C, Lee SS, Lee SJ. Feedback regulation via AMPK and HIF-1 mediates ROS-dependent longevity in Caenorhabditis elegans. Proc Natl Acad Sci USA. 2014; 111:E4458–67. 10.1073/pnas.1411199111 25288734 PMC4210294

[47] Wang Y, Branicky R, Noë A, Hekimi S. Superoxide dismutases: dual roles in controlling ROS damage and regulating ROS signaling. J Cell Biol. 2018; 217:1915–28. 10.1083/jcb.201708007 29669742 PMC5987716

[48] Van Raamsdonk JM, Hekimi S. Reactive Oxygen Species and Aging in Caenorhabditis elegans: Causal or Casual Relationship? Antioxid Redox Signal. 2010; 13:1911–53. 10.1089/ars.2010.3215 20568954

[49] Davalli P, Mitic T, Caporali A, Lauriola A, D’Arca D. ROS, Cell Senescence, and Novel Molecular Mechanisms in Aging and Age-Related Diseases. Oxid Med Cell Longev. 2016; 2016:3565127. 10.1155/2016/3565127 27247702 PMC4877482

[50] Jang JY, Blum A, Liu J, Finkel T. The role of mitochondria in aging. J Clin Invest. 2018; 128:3662–70. 10.1172/JCI120842 30059016 PMC6118639

[51] Swerdlow RH, E L, Aires D, Lu J. Glycolysis-respiration relationships in a neuroblastoma cell line. Biochim Biophys Acta. 2013; 1830:2891–98. 10.1016/j.bbagen.2013.01.002 23313167 PMC3594384

[52] Thurman RG, Scholz R. Interaction of glycolysis and respiration in perfused rat liver. Changes in oxygen uptake following the addition of ethanol. Eur J Biochem. 1977; 75:13–21. 10.1111/j.1432-1033.1977.tb11499.x 862614

[53] Loesberg C, Van Rooij H, Nooijen WJ, Meijer AJ, Smets LA. Impaired mitochondrial respiration and stimulated glycolysis by m-iodobenzylguanidine (MIBG). Int J Cancer. 1990; 46:276–81. 10.1002/ijc.2910460223 2384275

[54] Stincone A, Prigione A, Cramer T, Wamelink MM, Campbell K, Cheung E, Olin-Sandoval V, Grüning NM, Krüger A, Tauqeer Alam M, Keller MA, Breitenbach M, Brindle KM, et al. The return of metabolism: biochemistry and physiology of the pentose phosphate pathway. Biol Rev Camb Philos Soc. 2015; 90:927–63. 10.1111/brv.12140 25243985 PMC4470864

[55] Kuehne A, Emmert H, Soehle J, Winnefeld M, Fischer F, Wenck H, Gallinat S, Terstegen L, Lucius R, Hildebrand J, Zamboni N. Acute Activation of Oxidative Pentose Phosphate Pathway as First-Line Response to Oxidative Stress in Human Skin Cells. Mol Cell. 2015; 59:359–71. 10.1016/j.molcel.2015.06.017 26190262

[56] Cai Y, Wei YH. Distinct regulation of Maf1 for lifespan extension by Protein kinase A and Sch9. Aging (Albany NY). 2015; 7:133–43. 10.18632/aging.100727 25720796 PMC4359695

[57] Cai Y, Wei YH. Stress resistance and lifespan are increased in C. elegans but decreased in S. cerevisiae by mafr-1/maf1 deletion. Oncotarget. 2016; 7:10812–26. 10.18632/oncotarget.7769 26934328 PMC4905441

[58] Jia K, Thomas C, Akbar M, Sun Q, Adams-Huet B, Gilpin C, Levine B. Autophagy genes protect against Salmonella typhimurium infection and mediate insulin signaling-regulated pathogen resistance. Proc Natl Acad Sci USA. 2009; 106:14564–69. 10.1073/pnas.0813319106 19667176 PMC2731839

