# Supplementary material for: Conserved roles of glucose in suppressing reactive oxygen species-induced cell death and animal survival
Source: Aging (Albany NY). 2019 Aug 12;11(15):5726–43. doi: 10.18632/aging.102155 (PMC6710067; doi:10.18632/aging.102155)
Supplement: Supplementary Information [file aging-11-102155-s001.pdf]

SUPPLEMENTARY INFORMATION

ROS detection

For ROS detection in *C. elegans*, mitochondrial ROS was detected using the mitochondrial-localized ROS sensor Mitoracker (MitoTracker® Red CMXRos), which has also been described before [1]. Animals were synchronized by collecting eggs from gravid animals in a 2-hour time frame on nematode growth medium (NGM) agar plate (60 x 15 mm) with or without 0.5% glucose to L4 stag, then transferred to the same glucose plates added with 1mM paraquat a day before. After paraquat treatment for 24 hours, animals were washed with M9 buffer at least 5 times to remove bacteria food, which could potentially affect the mitotracker signals. Clean animals were then incubated with 5uM MitorackerRed in M9 buffer for 2 hours with gentle shaking to allow uptake of Mito-Tracker dye. Animals were then washed extensively to remove MitoTracker dye and further raised on nematode growth medium (NGM) agar plate for at least 4 hours before imaging with fluorescent microscope. For ROS detection in mammalian cells, NIH3T3 cells were seeded in six-well plates at 5×10^4 cells per well in low glucose DMEM. When cells reached 60% confluency, medium were changed to low (1mg/L), normal (4mg/L) and high glucose (8mg/L) with each supplemented with or without 100nM rapamycin. Cells were collected after 1, 2, 4, and 8 hours, washed with PBS and stained with 10 µM of fluorescent probe DCFH-DA (Beyotime Biotechnology,China) in serum-free RPMI-1640 medium at 37 °C for 30min. Cells were washed with PBS, and cellular ROS contents were measured using a CytoFLEX-S flow cytometer (Beckman Coulter, USA).

Hatching rate measurement

C. elegans gravid adult were transferred to NGM agar plate containing control NGM agar plate (60 x 15 mm) and those supplemented with either 1mM paraquat only, 1% of glucose only, or both. Gravid worms were allowed to lay eggs for 2 hours to obtain sufficient eggs (5 worms/plate, 3 plates for each sample). Gravid worms were then picked and removed from drug plate. Eggs were allowed to hatch and animals developed for about 4 days to reach adulthood. Adult animals were then picked on normal NGM agar plate (5 worms/plate) to lay synchronized eggs for 2 hours for hatching measurement. After egg laying, eggs were counted, then incubated at 20°C for 2 days. Hatching rate were calculated by dividing the number of live animals by the number of eggs.

Western blot

Western blot of worms crude lysate has also been described before [2]. *C. elegans* gravid worms (CF2189, *Is001*[Pskn-1::skn-1::GFP + rol-6(su1006)]) were picked to NGM agar plate (60 x 15 mm, 5 worms/plate) with or without 0.5% glucose for 2 hours to lay synchronized eggs (10 glucose plate an 10 control plate). Worms were then cultured at 20°C for 2 days to reach L4/young adult stage, then worms were washed form plates and half of the glucose-treated animals and half of the control animals added to NGM agar plate with 1mM paraquat. Other halves were cultured on regular NGM agar plate to serve as control. Worms from these treatments were then harvested 2 days later, washed extensively with ice-cold M9, then resuspended in ice-cold lysis buffer (50 mM HEPES, pH 7.4, 1 mM EGTA, 1 mM MgCl2, 100 mM KCl, 10% glycerol, 0.5% NP-40, 2mM PMSF, Roche protease Complete inhibitor cocktail and phosSTOP tablet). Worms were then sonicated 10 times, 5 second each time on ice with interval of 15 second at the power level of 30%. Crude lysate was obtained by centrifugation. Whole lysate was subjected to SDS-PAGE and transferred to PVDF membrane. Membranes were blocked in PBST (PBS with 0.1% Tween-20) containing 5% non-fat milk for 1 hour and probed with anti-GFP (Abcam, ab32146) and anti-actin antibodies (Abcam, ab14128) at 1000X dilution for 1 hour. Membrane was washed extensively with 0.1% PBST, then incubated with anti-rabbit or anti-mouse HRP-conjugated secondary antibodies diluted in 0.1%PBST at 10,000X for 30 min. membrane were then proved with enhanced chemiluminescence (ECL) chemicals developed on radiographic films.

Mammalian cell culture

NIH3T3 cells were obtained from the Shanghai Institutes for Biological Sciences, Chinese Academy of Sciences. Cells were maintained in DMEM (Sigma) supplemented with 10% FBS (Gbico) and 100 µg/mL Penicillin-Streptomycin Solution (Gbico) at 37 °C in a humidified atmosphere containing 5% CO2 with frequent medium change. Cells were allowed for at least 3 passages after thawing from nitrogen tank for any assay.

Cell viability assay

Cell viability assay was evaluated by Cell Counting Kit-8 (CCK-8) (Dojin Laboratories, Japan). Cells were plated and incubated in 96-well plates at a density of 2×10^3

cells/well at different glucose conditions (1g/L,4g/L, 8g/L). When reaching 60–70% confluence, cells were treated with different concentrations of paraquat for 24 h. Then the plates were incubated with 10ul CCK-8 for 60 min. Finally, absorbance was measured at 450nm through SpectraMAXi3x plate reader (Molecular Devices, Austria).

Apoptosis assay

Apoptotic cells were quantified using the Annexin V-fluorescein isothiocyanate (FITC) /propidium iodide (PI) apoptosis assay (KeyGen BioTECH,China). Briefly, cells were plated in 6-well plates at a density of 5×10^4 cells/well with norma(4g/L) and high(8g/L) glucose concentration. When the confluence was 60-70%, cells were treated with 500 µM paraquat for 24 h. Sub-sequently, adherent cells were collected and washed twice with cold PBS. Cells were resuspended in 500 µl of manufacturer-supplied 1X binding buffer. Then, 5 µl of Annexin V-FITC and 5 µl of propidium iodide (PI) were added and incubated for 15 min in the dark at room temperature.Lastly,the ratios of apoptotic cells were monitored by CytoFLEX-S flow cytometer (Beckman Coulter, USA).

Image quantification

For quantification of signal intensity of ROS and *gst-4::gfp*, images taken were applied to image J software, worm were outlined and the signal inside the outline were determined by software. The intensity was obtained by dividing the signal reads by the worm areas. 10 images for each sample from 3-independent experiments were chosen randomly for quantification and normalized to the average value of control group. For SKN-1 nuclear localization quantification, due to the variation in different part of the animal body, we count how many intestine cells show observable SKN-1::GFP signal. Images for 10 worms were randomly chosen and SKN-1:GFP positive nuclei were counted and plotted in Prism software.

Statistical analysis

Statistical study was conducted by using software Prism. Bar data were examined by student's t-test, with significant difference defined by $P < 0.05$. Survival curves were examined by Log-rank test, with significant difference defined by $P<0.05$.

REFERENCES

1. Wei Y, Kenyon C. Roles for ROS and hydrogen sulfide in the longevity response to germline loss in Caenorhabditis elegans. Proc Natl Acad Sci USA. 2016; 113:E2832–41. https://doi.org/10.1073/pnas.1524727113 PMID:27140632

2. Cai Y, Wei YH. Stress resistance and lifespan are increased in C. elegans but decreased in S. cerevisiae by mafr-1/maf1 deletion. Oncotarget. 2016; 7:10812–26. https://doi.org/10.18632/oncotarget.7769 PMID:26934328

3. Li X, Matilainen O, Jin C, Glover-Cutter KM, Holmberg CI, Blackwell TK. Specific SKN-1/Nrf stress responses to perturbations in translation elongation and proteasome activity. PLoS Genet. 2011; 7:e1002119. https://doi.org/10.1371/journal.pgen.1002119 PMID:21695230

4. Rudgalvyte M, Peltonen J, Lakso M, Wong G. Chronic MeHg exposure modifies the histone H3K4me3 epigenetic landscape in Caenorhabditis elegans. Comp Biochem Physiol C Toxicol Pharmacol. 2017; 191:109–16. https://doi.org/10.1016/j.cbpc.2016.10.001 PMID:27717699
