# Supplementary material for: Conserved roles of glucose in suppressing reactive oxygen species-induced cell death and animal survival
Source: Aging (Albany NY). 2019 Aug 12;11(15):5726–43. doi: 10.18632/aging.102155 (PMC6710067; doi:10.18632/aging.102155)
Supplement: Supplementary Figure 1 [file aging-11-102155-s002.pdf]

SUPPLEMENTARY FIGURE

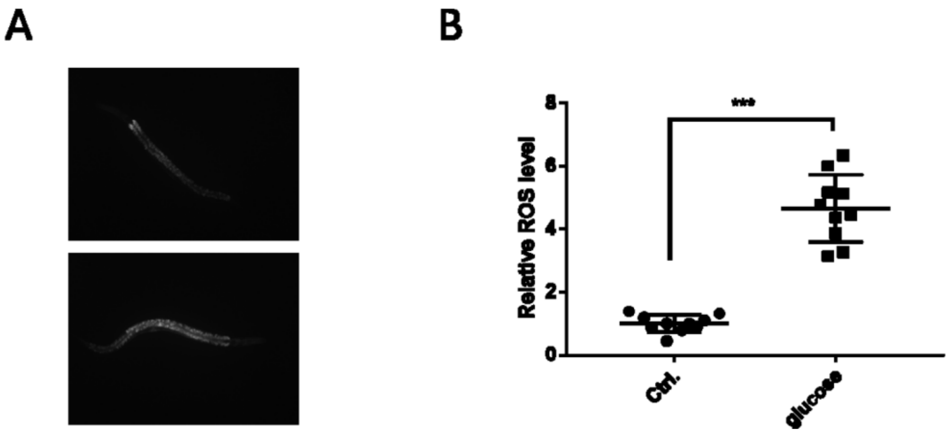

Supplementary Figure 1. Long term glucose treatment increases overall ROS in *C. elegans*. (**A**) Animals at day-10 of adulthood were incubated in M9 buffer containing 3 μM Dihydroethidium (DHE) for 30 min washed extensively with M9 buffer again before imaging. Representative images of 2 independent experiments were shown. (**B**) Quantification of DHE-stained ROS in day-10 adulthood by ImageJ. 10 images were randomly selected from 2-independent experiment and signal intensity were quantified. Reads were normalized to the average value of control (Ctrl) group and plotted as relative ROS levels. P value was derived from student's t-test: ***, P<0.0005.
