# Supplementary material for: Conserved roles of glucose in suppressing reactive oxygen species-induced cell death and animal survival
Source: Aging (Albany NY). 2019 Aug 12;11(15):5726–43. doi: 10.18632/aging.102155 (PMC6710067; doi:10.18632/aging.102155)
Supplement: Supplementary Tables [file aging-11-102155-s003.pdf]

SUPPLEMENTARY TABLES

Supplementary Table 1. *C. elegans* strains used in this study.

Strain	Description
N2	Wild-type strain originally isolated from mushroom compost near Bristol, England.
CF1903	*glp-1(e2144) III.*
MQ887	*isp-1(qm150) IV.*
CF2189	*Is001*[Pskn-1::skn-1::GFP + rol-6(su1006)]
CL2166	*dvIs19*[pAF15(*gst-4*::GFP::NLS)] III
CL2006	*dvIs2*[pCL12(*unc-54/*human Abeta peptide 1-42 minigene) + pRF4]
AM140	*rmIs132* [unc-54p::Q35::YFP]

Supplementary Table 2. Primer sets for RT-qPCR.

Gene	Forward primer sequence (5′-3′)	Reverse primer sequence (5′-3′)	Ref.
gst-4	CCCATTTTACAAGTCGATGG	CTTCCTCTGCAGTTTTTCCA	[3]
gst-5	GGTAAGAAGCTTGCTCAATC;	AATGCTGGAAGGAAGATGTC	[4]
gst-10	GTCTACCACGTTTTGGATGC	ACTTTGTCGGCCTTTCTCTT	[3]
gcs-1	AATCGATTCCTTTGGAGACC	ATGTTTGCCTCGACAATGTT	[3]
act-1	TCGGTATGGGACAGAAGGAC	CATCCCAGTTGGTGACGATA	[3]

Supplementary Table 3. Lifespan data in 4A.

Experiment	Genotype	Treatment	Death (censored)	Medium survival	P Values
1	N2 wild-type	ctrl	88(12)	22	<0.0001
	N2 wild-type	glucose	67(27)	16	
	isp-1(qm150)	ctrl	98(7)	28	<0.0001
	isp-1(qm150)	glucose	63(23)	16	
2	N2 wild-type	ctrl	98(10)	24	<0.0001
	N2 wild-type	glucose	87(31)	17	
	isp-1(qm150)	ctrl	93(14)	27	<0.0001
	isp-1(qm150)	glucose	82(36)	17	

Supplementary Table 4. Lifespan data in 4B.

Experiment	Genotype	Treatment	Death (censored)	Medium survival	P Values
1	N2 wild-type	ctrl	93(16)	24	<0.0001
	N2 wild-type	glucose	66(22)	16	
	glp-1(e2144)	ctrl	99(14)	30	<0.0001
	glp-1(e2144)	glucose	61(35)	16	
2	N2 wild-type	ctrl	78(30)	23	<0.0001
	N2 wild-type	glucose	57(44)	15	
	glp-1(e2144)	ctrl	82(23)	29	<0.0001
	glp-1(e2144)	glucose	72(31)	17	

Supplementary Table 5. Lifespan data in 4C.

Experiment	Genotype	Treatment	Death (censored)	Medium survival	P Values
1	N2 wild-type	ctrol	93(14)	21	<0.0001
	N2 wild-type	glucose	66(25)	15	
	N2 wild-type	PQ+ctrl	85(11)	27	<0.0001
	N2 wild-type	PQ+glucose	69(27)	15	
2	N2 wild-type	ctrl	85(16)	22	<0.0001
	N2 wild-type	glucose	57(39)	16	
	N2 wild-type	PQ+ctrl	86(22)	28	<0.0001
	N2 wild-type	PQ+glucose	64(40)	16	

PQ: paraquat (1mM), glucose
